# Introduction to special issue: floral ecology, genetics, and evolution in an unprecedentedly fast changing world

**DOI:** 10.1093/aobpla/plaf054

**Published:** 2025-09-29

**Authors:** F Xavier Picó, Anna Traveset, Mario Vallejo-Marin, Juan Arroyo

**Affiliations:** Departamento de Ecología y Evolución, Estación Biológica de Doñana (EBD), Consejo Superior de Investigaciones Cientíﬁcas (CSIC), 41092 Sevilla, Spain; Global Change Research Group, Mediterranean Institute for Advanced Studies, IMEDEA, CSIC-UIB, 07190 Esporles, Balearic Islands, Spain; Department of Ecology and Genetics, Evolutionary Biology Centre, Uppsala University, 752 36 Uppsala, Sweden; Department of Plant Biology and Ecology, Faculty of Biology, University of Seville, 41012 Seville, Spain

**Keywords:** citizen science, experiments, fieldwork, floral traits, droughts, heatwaves, mating systems, pollinators, pollen

## Abstract

The study of floral biology has long attracted the attention of plant biologists because of its enormous basic and applied implications, spanning from identification of the ecological and genetic drivers of flowering plant evolution to the performance of crop yields in agricultural systems. In a rapidly changing planet, floral biology studies acquire an utmost importance to comprehend the multiple ecological, economical, and social challenges ahead for humanity. In this special issue, we gathered a collection of papers dealing with various ecological, genetic, and evolutionary aspects of floral biology. This special issue encompasses 12 papers showcasing theoretical and empirical research on plant–pollinator communities, pollinators and pollination modes, floral ecology and genetics at various spatial scales, and the effects of warming-induced abiotic stress on floral biology. Overall, this special issue highlights the importance of long-term spatial and temporal studies, which require a collaborative effort of the research community, and the development of experimental approaches to quantify in detail the effects of human-induced abiotic stress, such as droughts and heatwaves, on plant reproduction.

## Teaser

This note introduces the collection of papers of the special issue entitled ‘Floral ecology, genetics, and evolution in an unprecedentedly fast changing world’. The papers of this special issue deal with theoretical and empirical research on plant-pollinator communities, pollinators and pollination modes, floral ecology and genetics at various spatial scales, and the effects of warming-induced abiotic stress on floral biology.

## Introduction

Floral ecology, genetics, and evolution are central to understanding how plants reproduce, interact with pollinators, and adapt to dynamic environments. Floral traits such as shape, colour, and floral rewards to pollinators, mediate ecological interactions, while genetic processes regulate reproductive strategies, gene flow, and population differentiation. Integrating these perspectives provides critical insights into the evolutionary mechanisms shaping biodiversity. In the context of rapid global change, involving habitat loss and fragmentation, climate change, biological invasions, and unprecedented pollinator shifts and declines, plant reproductive systems face increasing pressures that may alter both the structure and the dynamics of ecological plant–pollinator interactions, depicted as ecological networks, and evolutionary trajectories. Investigating how floral traits and genetic architectures respond to such disturbances is essential for predicting plant resilience, ecosystem stability, and the persistence of key ecological functions. Such knowledge is also vital for anticipating the impacts of global change on food security, as many crop yields are tightly linked to plant–pollinator dynamics.

This special issue commemorates the 20th anniversary (2004–23) of the EcoFlor working group, a network of biologists interested in floral ecology and evolution, sponsored by the Ecological Association of Terrestrial Ecology (AEET; https://www.aeet.org/es/) of Spain. The EcoFlor’s annual meetings bring together scientists and professionals interested in all aspects of floral ecology and evolution at both national and international levels. Since 2004, EcoFlor has attracted undergraduate, graduate, and senior researchers from various institutions, fostering interdisciplinary efforts for scientific discovery and providing an inspiring and welcoming environment for new generations of scientists. EcoFlor has grown rapidly since its foundation, which began gathering 15 people from Spanish institutions, to congregate today up to 100+ people from different countries every year. For example, the analysis of the scientific programmes between 2004 and 2025 indicated that co-authorships of all communications presented in EcoFlor annual meetings include 97 unique authors from 58 institutions based in 15 countries. The work presented at EcoFlor has dealt with multiple topics, with special emphasis on evolution of floral traits, pollinator ecology, plant mating systems and reproductive biology, population genetics and phylogenetics, plant–pollinator networks, ecological drivers of plant–animal interactions, conservation biology, and various conceptual and methodological advances for the study of floral biology. EcoFlor has also played an important training role for early career researchers through specific workshops on a diverse array of methodologies to address questions in floral biology. Overall, this special issue provides a snapshot of the 20+ years of EcoFlor history, but also of the fields of floral ecology, genetics, and evolution in a planet experiencing global changes at unprecedented rates.

## Plant–pollinator communities in a changing world

This special issue begins with three papers addressing the complexity of plant–pollinator communities from very different approaches. In a first paper, Magrach and Montoya (2024) review the literature on the study of ecological stability, i.e. the ability of an ecosystem to minimize the variability over time or to recover after a perturbation, in plant–pollinator communities, but centred on multiple trophic levels and considering the interactions that relate species to each other. The review clearly uncovers the limited knowledge available on ecological stability in plant–pollinator communities when considering multiple factors, as existing studies mostly address this issue partially. This result is not surprising, as the topic is extremely challenging and limitations are many. The authors emphasize the importance of extending studies of ecological stability in plant–pollinator communities to multi-year datasets across spatial scales, as this is critical for understanding complexity, and of incorporating multiple stability metrics alongside diverse ecological components of the community. The advances in the study of ecological stability in plant–pollinator communities at the level they proposed will not be possible without the long-term collaborative effort of the research community.

The second paper presents a rare opportunity to understand the long-term dynamics of plant–pollinator communities (Herrera 2019, 2020). To assess temporal variation in pollinator contributions to pollen transfer, Freudenfeld et al. (2025) report on pollen load across a diverse spectrum of insect pollinator taxa and variation in their abundance over 11 years from a semi-natural grassland in Central Bohemia. The authors generate a dataset, including ca. 45 500 pollinator visits to flowers, which highlight the importance of diverse and species-rich pollinator communities for the maintenance of the whole ecosystem. This is because pollen load varied little among pollinator taxa, indicating their fixed effectiveness, whereas their abundances fluctuated strongly over time. Consequently, each taxon’s contribution to pollen transfer depends largely on its abundance, indicating that a pollinator community can buffer declines in some species through compensatory increases in others. This paper focuses on the pollinator side of the plant–pollinator community, which is not common in the literature, rather than on flower traits, and thus makes a noteworthy contribution to the field.

In the third paper, Bitonto et al. (2025) present the first results of the LIFE 4 Pollinators project (https://www.life4pollinators.eu/en/submission) to validate the potential of a citizen science approach in studying plant–pollinator communities in wild environments, as also shown in urban areas (Mason and Arathi 2019). In agreement with the previous two papers, this study remains us of the need to consider large spatial and temporal scales to grasp the complexity and dynamics of plant–pollinator communities. Citizen science projects like this one complement the work of the research community. The results, including over 2000 photographs of plant–pollinator interactions in 4 years (2021–24) across four European countries, reveal the value of the data uploaded by the users, as they correctly identified ca. 94% of insect taxonomic aggregations and 74% of plant species. Despite some inevitable biases (geographic, taxonomic, uneven involvement of volunteers), these high percentages of correct insect and plant identifications allow authors to be optimistic about the important contributions of citizen-generated data for research, conservation, and management of natural areas. Nevertheless, the authors stress key points to increase the impact of this sort of initiatives, such as the value of citizen motivation and engagement over the whole process, the development of activities involving researchers and volunteers, or the availability of clear protocols to generate and upload the data, to name a few. Fine-tuning all these aspects will allow researchers to design and develop more ambitious citizen science projects to address specific goals with higher scientific and conservation relevance.

## Pollinators and pollination modes

Several papers of this special issue focus on pollinators and pollination modes because of their importance for plant reproduction, productivity, and evolution. For example, two papers in this section (Robles et al. 2024, Muñoz-Gallego et al. 2025) deal with pollination by vertebrates (birds and lizards) on islands, as insects are known to be underrepresented on islands compared to the mainland, which may represent an important evolutionary force in such particular ecosystems. These studies conduct observational and experimental work on Mediterranean shrubs from the Balearic Islands in western Mediterranean Basin, such as *Malva arborea* (Malvaceae; Robles et al. 2024) and *Withania frutescens* (Solanaceae; Muñoz-Gallego et al. 2025), to identify flower visitors and quantify their visitation frequency, and to characterize in detail the plants’ reproductive system. Both studies provide solid evidence indicating that vertebrates were the most frequent visitors for these two plant species. Given the current marked trend of declining fieldwork-based research and education in ecology (Soga and Gaston 2025), these studies are a welcome reminder of the value of natural history and fieldwork in plant–pollinator research, and in other eco-evolutionary disciplines, as they represent the basis to understand the ecology and evolution of plant breeding strategies. Furthermore, this approach can also unveil previously unknown aspects of plant reproduction complexity and diversity, as illustrated by the discovery of the mixed sexual system in *W. frutescens*, which combines hermaphroditism with cryptic dioecy (Muñoz-Gallego et al. 2025).

Turning to insects as the main pollinators in flowering plants, two additional papers of this section address two very different issues in insect-based pollination biology. In the first one, Gómez et al. (2025) dig into the concept of pollination effectiveness. This trait refers to the contribution of one pollinator to the plant’s fitness at both quantitative (e.g. amount of pollen grains deposited by one pollinator or the number of visits per plant and time unit) and qualitative (e.g. the number of seeds produced per visit by one pollinator) levels. The authors explore the components of pollination effectiveness in *Moricandia arvensis* (Brassicaceae), a mustard widely distributed across arid regions of the Iberian Peninsula, which shows remarkable intra-individual floral polyphenism, with large cross-shaped lilac flowers during spring and small rounded white flowers during summer. Variation in pollination effectiveness between spring and summer allows the authors to conclude that the spring pollinator assemblage was more specialized in the former, stressing the value of effectiveness to characterize the architecture of plant–pollination interactions. This research paves the road for other studies on further qualitative components, such as the vigour and success of seeds produced.

In a second paper, Pérez-Barrales et al. (2025) evaluate the implications for a plant of flowering solo or co-flowering with congeneric species, which may alter the entire pollination environment of plants, including changes in pollen delivery and deposition patterns. The authors investigate this issue on *Palicourea coriacea* (Rubiaceae) in the Brazilian Cerrado during two flowering periods: early in the season, when flowering occurred alone, and later, when it overlapped with its conspecific *P. officinalis*. Integrated analyses of flowering phenology, floral morphology, and floral visitors provided strong evidence of significant reproductive interference between the two species in congeneric patches, despite phenological and morphological differences between species that would otherwise reduce the potential costs of pollinator sharing.

The last paper of this section by Grillo and Gutiérrez (2025) is rather unique as it deals with the specific floral traits underlying the mechanisms driving mating system variation in wind-pollinated plants and, in particular, how outcrossing can transition to selfing in plants that do not need pollinators to reproduce sexually. Given the massive amount of literature focused on animal–pollinated plants, this paper contributes to bridge an important gap of knowledge in the discipline (but see Friedman and Barrett 2009). Grillo and Gutiérrez (2025) work with accessions of two recently diverged sister grasses (*Oryza rufipogon* and *O. nivara*, Poaceae), both wind-pollinated, but one being an outcrosser (*O. rufipogon*) and the other a selfer (*O. nivara*). After considering several floral traits, the authors conclude that two traits emerge as the most important to provide reliable autogamous selfing in grasses, namely large basal pore of the anther and early dehiscence function. By experimentally forcing these two grasses to self-fertilize under different pollen availability treatments, the authors show that autogamous selfing is massive in the selfer *O. nivara*, whereas the outcrosser *O. rufipogon* achieves selfing through geitonogamy, highlighting functional divergence between the two sister species.

## Floral ecology and genetics at small and large spatial scales

A third group of papers focuses on identifying the ecological and genetic correlates of plant species differing in a wide diversity of floral and reproductive attributes, spanning from floral polymorphisms to various mating systems and pollination modes. Two of the four studies of this section develop comprehensive population-based approaches at regional and local scales and combine environmental data with genetic data obtained from co-dominant markers (e.g. simple sequence repeats), which provide the elements needed to grasp the micro-evolutionary history of study species. On the one hand, Vanrell et al. (2024) explore the ecological and genetic diversity of the wild flax complex (*Linum suffruticosum s.l.*, Linaceae). This is a polyploid complex of perennial woody plants with three-dimensional heterostylous white flowers and an obligate outcrossing mating system. The authors seek reproductive (morph ratio and sex organ reciprocity), biological (cytotype and taxonomic entity as a surrogate of morphological variation), demographic (population size), geographic (latitude and elevation), and environmental (climate and soil) drivers of genetic diversity and differentiation in more than 30 populations across the western Mediterranean Basin. This comprehensive regional study indicates that ploidy level (2×, 4×, 6×, and 8×) and distinctive geographical barriers to gene flow and migratory routes (North Africa and the Iberian Peninsula) emerge as the most importance forces shaping genetic diversity patterns in wild flax, whereas taxonomic entities did not account for genetic diversity or differentiation patterns. Studies like this one on plant species complexes with an active evolutionary history, which are common across the region (Agudo et al. 2023), provide clues to resolve the taxonomy of challenging plant groups.

Similarly, Heuertz et al. (2025) apply a micro-evolutionary approach at a local scale in a Mediterranean snapdragon (*Antirrhinum charidemi*, Plantaginaceae), a perennial shrub endemic to south-eastern Spain, thereby enabling the study of fine-scale drivers of incipient evolutionary differentiation. In this particular case, the study population of *A. charidemi* includes individuals differing in corolla colour (pink and white), topography (40-m altitude difference), substrate, and vegetation. Although most flower visitors exhibited a preference for pink-flowered individuals, along with a significant genetic differentiation between pink- and white-flower bearing individuals, the results clearly indicate that fine-scale spatial isolation in a topographically heterogeneous environment is the strongest driver of small-scale genetic differentiation in this snapdragon. Together, these two studies at regional and local scales demonstrate that changes in connectivity via migration and/or gene flow among individuals and populations seem to be the major forces in shaping genetic differentiation in plants, which represents the first step towards higher levels of diversification.

## Effects of warming-induced abiotic stress on floral biology

Finally, this special issue touches on the effect of two closely related natural hazards in a warming world on plants, such as droughts and heatwaves. Both extreme weather events are becoming increasingly perceptible, as its frequency and intensity are clearly on the rise in all continents (Zhang et al. 2024). The two studies conduct sophisticated experimental approaches to quantify how warming-induced abiotic stresses, such as water limitation by increased droughts and heat damages during heatwaves, affect key fitness-related floral traits. Their findings, broadly applicable to other plant systems, enhance our understanding of how extreme weather events may influence the evolution of wild plant species and affect crop yields in agricultural systems.

In a first paper, García et al. (2023) simultaneously test the effects of experimental reduction in pollinator access and water availability on floral signals (floral size and petal brightness) and nectar rewards (nectar volume and concentration) in the common morning glory (*Ipomoea purpurea*, Convolvulaceae). Their evolutionary approach links resource limitation directly to phenotypic selection on floral traits, an aspect rarely covered for nectar rewards. The study reveals that water deficit, not limited pollinator access, alters selection patterns. In particular, the common morning glory exhibits larger flowers and less nectar under drought, whereas well-watered plants show increased nectar volumes regardless of pollinator access. These findings highlight the evolutionary relevance of abiotic (water-related) stress over biotic factors on key floral traits, which may further affect patterns of pollinator visitation. Overall, the authors stress the value of using experimental approaches to uncover the relative role of abiotic and biotic agents of selection acting on multiple floral traits.

In the second paper, Rosenberger et al. (2024) undertake a hand-pollination experiment on developing flowers of the mustard *Brassica napus* (Brassicaceae) exposed to control temperature and extreme heat over a few days to test the effect of heatwaves on pollen traits (number of pollen grains, pollen viability and vigour). Specifically, they quantitatively and qualitatively evaluate the effects of extreme, prolonged heat on pollen, which has strong implications for wild plant populations and fruit-bearing crops. The results are clear as heat-treated plants exhibit reductions of ∼20% in pollen production during flower development, of ∼75% in pollen tube growth, and of >85% in seed set. The study also reveals that extreme heat even nullifies the benefits of cross-pollination for pollen tube survival in comparison to self-pollination, suggesting multiple eco-evolutionary implications, such as increased rates of inbreeding depression in wild populations and crops. The imposed heat treatment was 35°C over 3 days, while current heatwaves frequently exceed 40°C, lasting several days or even a few weeks in several world regions. Thus, our understanding of how heatwave frequency, intensity, and duration disrupt pollen quantity and quality, and thereby threaten reproduction in wild plants and agricultural crops, is just beginning to emerge (López et al. 2022).

## A way forward

Based on the collection of papers of this special issue on floral ecology, genetics, and evolution in an unprecedentedly fast changing world, we envisage some conceptual and technical challenges for the consolidation and advance of the discipline in the future. First, the need for long-term, widespread collaborative research that can only be undertaken with the involvement of the research community at large. Studying critical aspects, such as the long-term dynamics of plant–pollinator assemblages in wild environments and their ecological and evolutionary implications, are best tackled by large-scale, long-term studies. Second, the involvement of other societal actors in floral biology studies seems to be unavoidable, not only to increase awareness of floral biology among the public, but also to address more ambitious, complex, and valuable scientific goals beyond the reach of individual researchers or laboratories, leading to an unprecedented task force. Third, we need to underscore the importance of fieldwork in plant–pollinator research, especially given the current marked trend of declining fieldwork-based research and education in ecology. The increasing shortage of such knowledge obtained from the observation and quantification of patterns taking place in the wild may jeopardize future studies on plant breeding strategies, pollination effectiveness, reproductive interference, flowering phenology, and flowering plant evolution. Finally, the design of experimental approaches to quantify the response of plants to the current threats imposed by a changing climate, such as droughts and heatwaves, is crucial if we really aim at understanding how extreme weather events influence the evolution of wild plant species and affect crop yields in agricultural systems, for the safety of our own species. Ultimately, this kind information is critical for conservation of species and ecological systems. We hope that regular meetings on floral ecology, genetics, and evolution, such as Ecoflor or SCAPE (Scandinavian Association for Pollination Ecology), will continue contributing to the growth and development of the research community for many years.

## Data Availability

Not applicable.
